# Identification of novel genetic loci and candidate genes for progressive ethanol consumption in diversity outbred mice

**DOI:** 10.1038/s41386-024-01902-6

**Published:** 2024-06-29

**Authors:** Kristin M. Mignogna, Zachary Tatom, Lorna Macleod, Zachary Sergi, Angel Nguyen, Marie Michenkova, Maren L. Smith, Michael F. Miles

**Affiliations:** 1 Department of Human and Molecular Genetics, Virginia Commonwealth University, Richmond, VA USA; 2 Department of Pharmacology and Toxicology, Virginia Commonwealth University, Richmond, VA USA; 3 Department of Neurology, Virginia Commonwealth University, Richmond, VA USA; 4 VCU Alcohol Research Center, Virginia Commonwealth University, Richmond, VA USA

**Keywords:** Addiction, Behavioural genetics

## Abstract

Mouse behavioral genetic mapping studies can identify genomic intervals modulating complex traits under well-controlled environmental conditions and have been used to study ethanol behaviors to aid in understanding genetic risk and the neurobiology of alcohol use disorder (AUD). However, historically such studies have produced large confidence intervals, thus complicating identification of potential causal candidate genes. Diversity Outbred (DO) mice offer the ability to perform high-resolution quantitative trait loci (QTL) mapping on a very genetically diverse background, thus facilitating identification of candidate genes. Here, we studied a population of 636 male DO mice with four weeks of intermittent ethanol access via a three-bottle choice procedure, producing a progressive ethanol consumption phenotype. QTL analysis identified 3 significant (Chrs 3, 4, and 12) and 13 suggestive loci for ethanol-drinking behaviors with narrow confidence intervals (1–4 Mbp for significant QTLs). Results suggested that genetic influences on initial versus progressive ethanol consumption were localized to different genomic intervals. A defined set of positional candidate genes were prioritized using haplotype analysis, identified coding polymorphisms, prefrontal cortex transcriptomics data, human GWAS data and prior rodent gene set data for ethanol or other misused substances. These candidates included *Car8*, the lone gene with a significant cis-eQTL within a Chr 4 QTL for week four ethanol consumption. These results represent the highest-resolution genetic mapping of ethanol consumption behaviors in mice to date, providing identification of novel loci and candidate genes for study in relation to the neurobiology of AUD.

## Introduction

Alcohol use disorder (AUD) poses a significant global healthcare burden, contributing yearly to around 3 million deaths worldwide [1]. In the United States, 140,000 people die yearly from causes related to alcohol use, making it the third-largest cause of preventable death [2]. Genetic factors are thought to play a significant role, with twin studies routinely estimating ~50% heritability in the risk for AUD [3].

Considerable progress in recent large human genome-wide association studies (GWAS) has identified a growing number of genetic loci associated with alcohol consumption [4, 5], dependence [6, 7], AUD [8], and problematic alcohol use [9]. Animal models complement human genetic studies by providing improved control over environmental variance and dependent variables, allowing for mechanistic and direct candidate gene identification studies infeasible in humans. Mouse models have identified broadly-applicable biological mechanisms underlying specific behaviors associated with AUD including ethanol consumption [10], preference [11, 12], and withdrawal [13]. Intermittent ethanol access (IEA) procedures have been shown to model escalation of alcohol consumption as seen in the early stages of AUD [14, 15]. IEA consumption varies across different mouse lines, suggesting that genetic variance impacting progressive consumption can be studied in such models [16, 17]. Additionally, rodent models have identified brain regions functioning in the transition to compulsive consumption, such as prefrontal cortex and nucleus accumbens [18, 19]. Medial prefrontal cortex (mPFC) has been implicated in molecular and neurocircuitry plasticity underlying dependence-like consumption and cue-induced reinstatement in rodent models [19, 20].

Behavioral quantitative trait loci (QTL) studies in rodents have been used to implicate genetic loci modulating ethanol behaviors, but generally with broad confidence intervals ( > 10–40 Mbp) confounding candidate gene identification [21–23]. Recent development of rodent genetic models with increased genetic diversity and recombination have greatly improved QTL analysis [24–26]. Diversity Outbred (DO) mice, derived from random outbreeding of 8 genetically and phenotypically diverse founder strains [27], provide high levels of allelic variation and recombination events for high-resolution genetic mapping [25]. The progenitor strains exhibit marked diversity in ethanol-drinking phenotypes [16, 17] and DO mice have recently been used to identify genetic loci for ethanol sensitivity and having narrow confidence intervals ( ~ 4 Mbp) [28].

Here we describe the first use of DO mice and an IEA procedure to map behavioral QTLs (bQTLs) associated with ethanol consumption. Our results show significant bQTLs on multiple chromosomes with narrow support intervals. Candidate genes were then prioritized by a multi-step strategy using haplotype analysis, integration of DO mouse transcriptome data from medial prefrontal cortex, and bioinformatics studies including merging of genetic data on AUD and other animal model data on ethanol. Our results identify novel candidate genes and biological mechanisms for modulation of ethanol consumption behaviors. The work may both validate and broaden existing human studies on the genetics of alcohol consumption and AUD, while also providing novel hypotheses for future studies on the neurobiology of ethanol in rodent models.

## Methods

Full details on Methods and Materials are provided in Supplemental Materials. Additional data supporting these studies are provided on the Open Science Framework (https://osf.io/tfrzg/?view_only=232cfcc8d9a94d16846138f64e4e3e59)

### Ethics statement

All animal care and euthanasia procedures were performed in accordance with the rules and regulations established by the United States Department of Agriculture Animal Welfare Act and Regulations, Public Health Services Policy on Humane Care and Use of Laboratory Animals, and American Association for Accreditation of Laboratory Animal Care. Humane endpoints were established by the same standards.

### Animal studies

Male DO mice (*n* = 636) were acquired from Jackson Laboratories after weaning at 4–6 weeks of age in 7 cohorts (average *n* = 106) spanning DO generations 22–25. Mice were singly housed in temperature- and humidity-controlled vivariums on cedar shaving bedding with *ad libitum* access to water and standard chow (#7912, Harlan Teklad, Madison, WI, United States). Sample size considerations and exclusion of female mice were chosen to maximize power to detect significant bQTL and are described in in Supplemental Materials. Study of ethanol consumption in ~600 animals was predicted to have 80% power to detect alleles causing less than 5% variance in a trait at a *p* value of <0.05 [29].

### Intermittent ethanol access

**Fig. 1:**
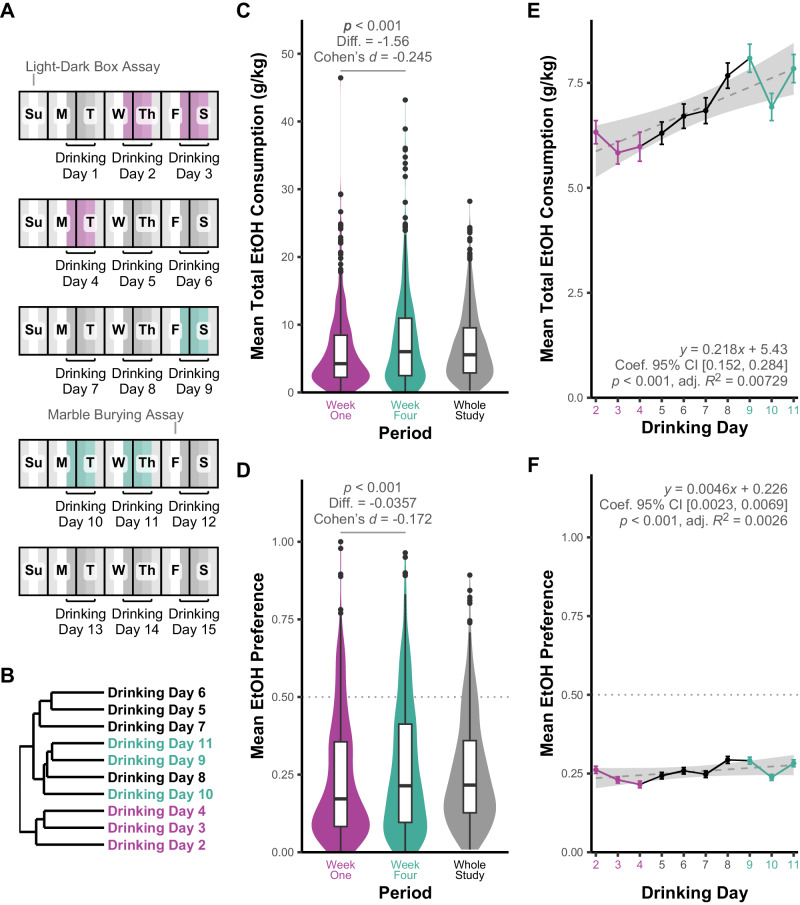
Diversity Outbred mice demonstrate progressive increase in ethanol consumption over four weeks of intermittent ethanol access. Mice were given voluntary access to ethanol via a three-bottle choice (H2O, 15% EtOH, 30% EtOH) procedure for 24 hours, starting at the beginning of each dark cycle (**A**). Hierarchical clustering of daily ethanol consumption identified two superclusters, one including drinking days 2–4 and one including drinking days 5–11 (**B**). Week four mean ethanol consumption was significantly higher than week one mean consumption in a within-sample one-tailed t-test (**C**). Drinking day was a significant predictor of ethanol consumption in linear regression, with consumption increasing by approximately 0.2 g EtOH/kg body weight each successive day (**E**). Similarly, week four ethanol preference was higher than week one ethanol preference (**D**) and drinking day was a significant predictor of ethanol preference, explaining a small proportion of overall variance (**F**).

Mice (*n* = 587) were studied for ethanol consumption via a three-bottle choice (15% ethanol v/v, 30% ethanol v/v, and water) IEA procedure for 5 weeks with alternating 24-hour periods of ethanol access (Monday, Wednesday, Friday) (Fig. 1A). 49 additional animals were exposed to only water as controls for anxiety-related behavioral studies and RNAseq analyses. Only the first 4 uninterrupted weeks of IEA were used for the purposes of behavioral genetic studies in this report, excluding day 1 of IEA due to increased variance on initial exposure to ethanol bottles. As described in Supplemental Materials, a marble-burying assay was done after week 4 of IEA as a study on abstinence-induced anxiety. This data and that from a basal light-dark box assay done before IEA exposure are not discussed in this report. However, an additional week of IEA was conducted after the marble burying assay to re-establish consumption levels prior to tissue harvesting.

### Tissue sample collection and genotyping

Mice were euthanized 24 hours after the end of their last ethanol exposure period via cervical dislocation and decapitation and tissue samples were collected immediately afterwards, flash-frozen in liquid nitrogen and stored at −80 °C. Brains were microdissected into nine regions as previously described [30, 31]. Tail snips were collected for genotyping (NeoGen Inc; Lincoln, NE) using a GigaMUGA microarray (*n*_SNP_ = 141,090; *n*_CNV_ = 2169) designed to optimize genotyping of DO mice [32] Of the initial 636 DO mice, full datasets were only collected from 630 mice as 6 mice reached humane endpoints before the end of experimentation. Data cleaning removed an additional 29 mice (22 for poor genotyping quality and 7 for potential sample mix-ups), resulting in a final sample of 603 mice (554 ethanol-drinking mice and 49 ethanol-naïve controls) used for analyses presented here.

### Behavioral QTL analysis

QTL mapping was carried out using the R/qtl2 software package. Dependent variables included whole study (drinking days 2–11), week one (days 2–4), and week four (days 9–11) averages for ethanol consumption (g ethanol/kg/24 h), ethanol preference (total ethanol consumed in ml/total fluid consumed in ml), and 30% choice (ml 30% ethanol/ml total ethanol) [33]. A week 4–week 1 difference in total ethanol consumption was used as an additional phenotype. Cohort was included as a fixed-effect covariate in all our analyses and kinship between mice was calculated using a linear mixed model and the leave-one-chromosome-out method. Phenotypes were either log- (for consumption and 30% choice) or square-root-transformed (for ethanol preference) to obtain normality before running analyses. Logarithm of the Odds (LOD) scores were calculated for each marker and permutation analysis (*n*_perm_ = 1000) used to calculate genome-wide empirical *p* values. SNP variant LOD scores were similarly calculated for QTL intervals and significance level *p* values determined by permutation across the involved chromosome. To detect founder strain effects, haplotype analysis was conducted for chromosomes containing significant or suggestive bQTLs using best linear unbiased predictors (BLUPs) in R/qtl2.

### Expression QTL analysis

To further implicate positional candidate genes from bQTL intervals, we utilized an existing expression QTL (eQTL) analysis of medial prefrontal cortex (mPFC) samples from a subset of 220 DO mice chosen for RNA-seq analysis based on average total ethanol consumption during the fourth week of IEA [34]. mPFC was chosen as a high-priority brain region due to its known role in decision-making, ethanol consumption and reinstatement and functional changes resulting from ethanol exposure as described in the Introduction. This included 100 mice from each extreme of the ethanol consumption distribution and 20 ethanol-naïve control mice. Detailed methodology for RNA-seq data, including alignment to individual genomes using Genotype-by-RNA-Seq (GBRS) [35], is described in Supplemental Methods and is reported elsewhere [34]. GBRS count data was used to generate eQTLs and haplotypes thereof within R/qtl2. Empirical significance of cis-eQTLs were determined by permutation analysis as described in Supplemental Methods and are defined as suggestive (*p* < 0.63, LOD ≥ 6.13) or significant (*p* < 0.05, LOD ≥ 8.45).

## Results

### DO mice exhibit variable patterns of initial versus progressive increase in ethanol consumption over time

Following exclusion of select mice as detailed in Methods, the remaining population of 554 ethanol-exposed DO mice exhibited wide variation in ethanol consumption, preference, and 30% choice over 4 weeks of IEA (Figs. 1 and S1) with ethanol consumption varying from <1 g/kg/24 h to >35 g/kg/24 h in week four of consumption. Consumption increased over time (Fig. 1E) and ethanol intake in week four was significantly higher than in week one (Fig. 1C, *p* = 1.006 × 10^−05^). Week one and week four consumption were significantly correlated (0.58, *p* = 1.08×10^-52^). Preference was significantly positively correlated with ethanol consumption across all time periods (*p* < 0.05) and increased significantly from week one to week four (Fig. 1D). 30% choice was not significantly correlated with ethanol consumption across all time periods, suggesting that mice preferring 30% ethanol did not necessarily increase ethanol intake overall (Fig. S2).

Hierarchical clustering of the ethanol consumption time course identified two major clusters, one containing drinking days 2–4 and one containing drinking days 5–11 (Fig. 1B). Principal component analysis of total daily ethanol consumption showed one principal component explained a large percentage (42.4%) of the variance in daily ethanol consumption (Fig. S3A) and was evenly positively loaded across all drinking days (Fig. S1B) and correlated with whole study mean total ethanol consumption on biplot analysis (Fig. S3E). In contrast, a second principal component loaded positively with week 1 drinking and negatively with drinking days 8–11 (Fig. S3B). This component also showed similar results in biplot analysis, being positively loaded with week one mean ethanol consumption (Fig. S3C) and negatively with week four mean ethanol consumption (Fig. S3D). While the first principal component may influence a major portion of overall ethanol consumption, the second component appears to explain variance between week one and week four consumption. This suggests possible genetic factors controlling initial consumption versus escalation in consumption over time, consistent with the hierarchical clustering data. We therefore chose to separately analyze week one and week four of ethanol access in addition to whole study ethanol drinking phenotypes for genetic mapping. The difference between week 4 and week 1 consumption was also studied to assess possible genetic influences on escalation.

SNP-based heritability estimates for ethanol consumption ranged from 0.197 to 0.310, and values for ethanol preference ranged from 0.143 to 0.301. Generally, week one heritability estimates were lower than for either last week or whole study. Estimates for 30% choice were much lower, ranging from 0.00 to 0.073 (Table S1).

### bQTL analysis identifies 3 significant loci and multiple suggestive loci for ethanol intake

**Fig. 2:**
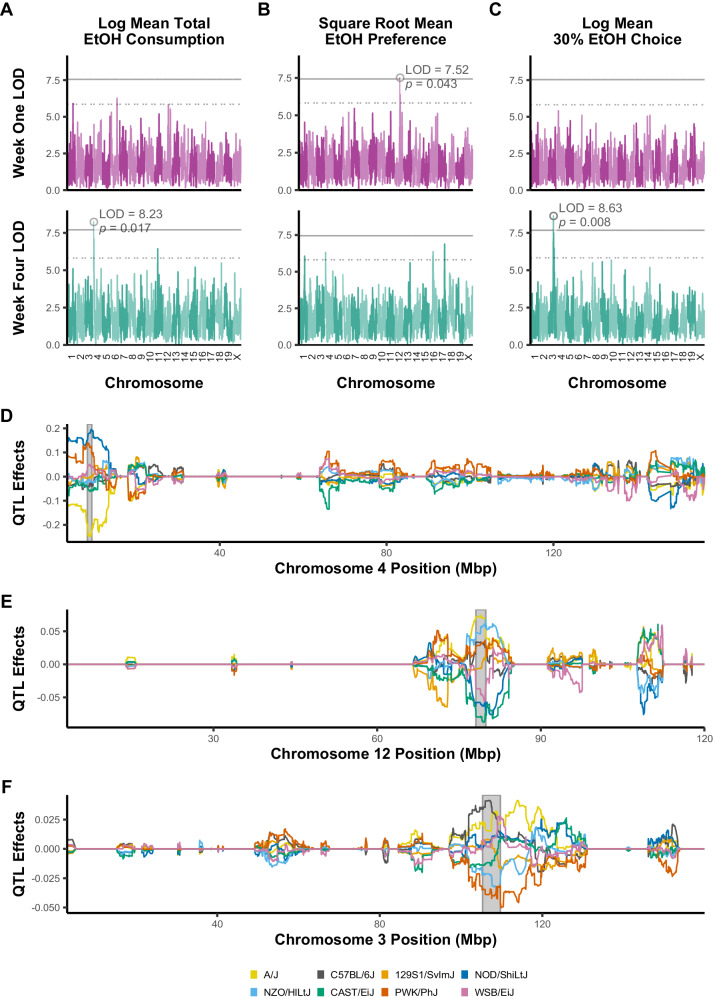
QTL analysis identifies significant peaks for three ethanol-related behavioral phenotypes. Different genetic effects were identified for week one and week four ethanol consumption (**A**), preference (**B**), and 30% choice (**C**) behavioral QTLs. For ethanol consumption, no significant QTL was observed during week one of the study, but a significant QTL on Chromosome 4 was identified in week four (LOD = 8.23; *p* = 0.017). For ethanol preference, a significant QTL was observed on Chromosome 12 (LOD = 7.52, *p* = 0.043) during week one of the study. For 30% choice a significant QTL was identified on Chromosome 3 during week four (LOD = 8.63, *p* = 0.008). Empirical significance thresholds for each phenotype were calculated using permutation analysis (n_perm_ = 1000); solid black lines represent *p* < 0.05 and dashed black lines represent *p* < 0.63 thresholds. **D**–**F** Haplotype analysis was carried out using best linear unbiased predictors for each significant bQTL. Support intervals for bQTLs are grayed. A/J alleles (yellow) contributed negatively to week four ethanol consumption (**D**) and positively to week one ethanol preference (**E**). PWK alleles (red) contributed negatively and C57BL/6 J (gray) contributed positively to week four 30% choice (**F**).

Significant bQTLs (*p* < 0.05) were identified on Chromosome (Chr) 4 for week four mean ethanol consumption (W4_EC; LOD = 8.23), on Chr 3 for week four mean 30% choice (W4_30C; LOD = 8.63), and on Chr 12 for week one mean ethanol preference (W1_EP; LOD = 7.52) (Fig. 2). An additional 13 suggestive QTLs (*p* < 0.63) were identified, and 95% Bayesian credible intervals were estimated for all QTLs (Table 1). All significant and suggestive bQTLs explained between 4% and 6% of observed variance in their relevant phenotypes, with significant bQTLs identifying greater genetic variance (Table 1). No significant or suggestive QTLs were found for the drinking phenotype principal components described above (Figs. S3F and S3G). The week 4 – week 1 difference analysis identified a suggestive QTL on Chr 15 overlapping with a week 4 ethanol preference suggestive QTL in that location (Fig. S7).

**Table 1:** Suggestive and significant QTLs for ethanol consumption behaviors.

Phenotype	LOD Score	*p* value	Chr	Peak Marker Position (Mbp)	95% Bayesian C.I. (Mbp)	# of Genes in C.I.	% Variance Explained	Previously-Published Substance Use QTL
Week Four Mean 30% Choice (W4_30C)	8.63	0.008	3	108.23	105.92–109.69	149	7.13	[11, 37, 52, 53]
Week Four Mean Ethanol Consumption (W4_EC)	8.23	0.017	4	8.41	8.24–9.29	20	6.8	
Week One Mean Ethanol Preference (W1_EP)	7.52	0.043	12	79.35	78.09–79.91	44	6.25	[54]
Week Four Mean 30% Choice (W4_30C)	6.93	0.146	3	103.57	101.85–104.47	96	5.77	[11, 37, 52, 53]
Week Four Mean Ethanol Preference (W4_EP)	6.88	0.16	17	74.32	72.91–75.21	55	5.72	
Whole Study Mean Ethanol Consumption	6.67	0.217	4	8.32	3.78–9.65	84	5.56	
Whole Study Mean Ethanol Preference	6.65	0.227	1	135.35	134.64–136.59	70	5.54	
Week Four Mean 30% Choice	6.48	0.278	3	118.93	118.39–119.81	19	5.41	[11, 37, 52, 53]
Week Four Mean Ethanol Consumption	6.46	0.294	11	50.58	49.96–51.52	62	5.38	[55]
Week Four Mean Ethanol Preference	6.36	0.335	15	44.69	39.43–45.68	89	5.3	[56]
Week Four Mean Ethanol Preference	6.32	0.351	4	8.32	3.37–11.05	123	5.27	
Whole Study Mean Ethanol Preference	6.3	0.362	4	7.00	3.37–8.32	73	5.26	
Whole Study Mean Ethanol Consumption	6.29	0.363	11	70.61	69.86–71.34	104	5.25	[55]
Week One Mean Ethanol Consumption	6.27	0.378	6	113.92	112.74–115.31	90	5.23	
Week Four – Week One Mean Ethanol Consumption	6.20	0.445	15	48.62	45.66–50.27	12	4.74	[56]
Week Four Mean Ethanol Preference	6.06	0.47	1	134.73	119.53–144.91	544	5.05	

QTLs were identified for week one, week four, and whole study mean ethanol consumption, preference for ethanol compared to water (“ethanol preference”), preference for 30% ethanol compared to 15% ethanol (“30% choice”) and difference in consumption between week 4 and week 1 (week 4 – week1). QTLs are in descending order by LOD score.

Haplotype analysis within R/qtl2 implicated strain-specific allelic contributions to variation in ethanol traits at the significant bQTLs. For the significant Chr 4 W4_EC locus, A/J alleles were associated with lower consumption, whereas NOD and PWK alleles were associated with higher ethanol intake in week 4 (Fig. 2D). The Chr 3 W4_30C locus had A/J, C57BL/6 J, and WSB/EiJ alleles correlated with increased in 30% choice whereas PWK/PhJ alleles associated with a decrease in this phenotype (Fig. 2F). NZO/HlLtJ and A/J alleles at the significant Chr 12 locus were associated with an increase in W1_EP, while CAST/EiJ, NOD/SHiLtJ, and WSB/EiJ alleles showed decreased ethanol preference (Fig. 2I).

### Top variants identified within significant bQTL intervals

**Fig. 3:**
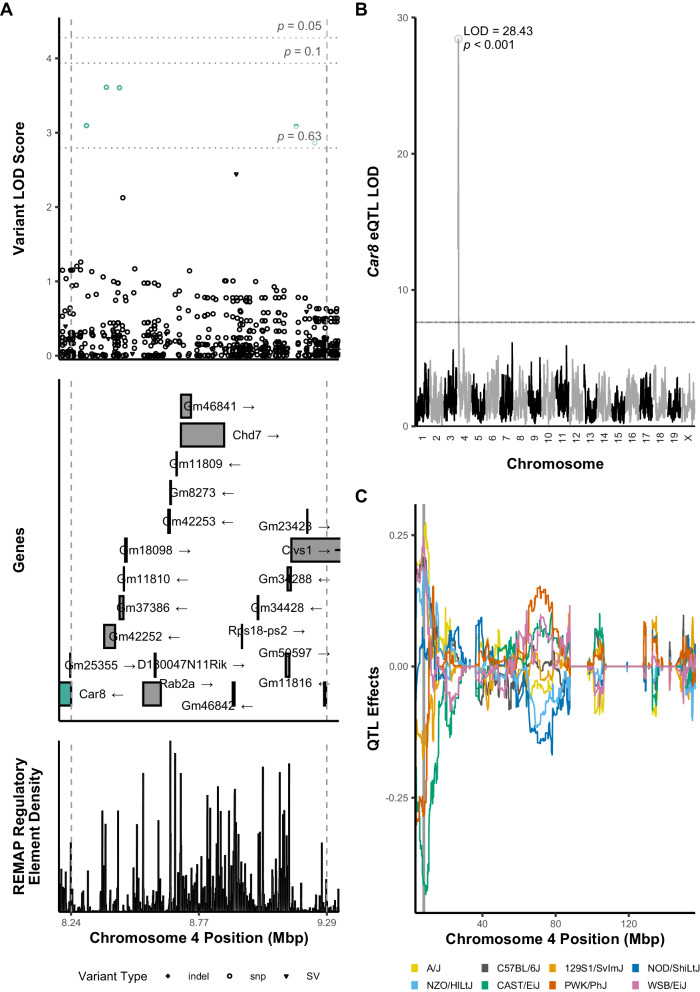
Variant LOD scores across significant bQTL on chromosome 4 for last week mean ethanol consumption. **A** SNP associations were estimated across the 95% Bayesian confidence interval identified for the significant bQTL, identified by dashed vertical lines (upper panel). Statistical thresholds identified by permutation analysis are indicated. Top variants were selected as those with a LOD score within 1.5 of the highest score as is conventional (Supplemental Table 2). Variants having suggestive *p* value significance for association with last week ethanol consumption are indicated (green). Known gene transcript annotations within this C.I. (middle panel) include *Car8, Rab2a*, *Chd7*, and *Clvs1*. As a highly ranked candidate gene (Table 2), *Car8* is colored (green). Lower panel indicates ReMap regulatory element density across the interval. **B** eQTL analysis for *Car8* indicates a highly significant *cis-*eQTL. **C** Haplotype analysis of *Car8* eQTL indicates A/J alleles (yellow) contribute positively while NOD alleles contribute negatively to expression of this gene.

Top SNPs within significant QTL confidence intervals were identified using a 1.5 LOD-drop from the peak SNP and permutation analysis (*n*_perm_ = 1000) to calculate empirical *p* values for variant LOD scores (Figs. 3A, S4 and S5). For the Chr 4 W4_EC QTL, the top three variants were intergenic SNPs located upstream of the *Car8* gene and downstream of predicted gene *Gm37386*, with the highest LOD score at rs249655952 (LOD = 3.84) (Fig. 3A, Table S2). Among 7 variants within a 1.5 LOD drop, 5 were unique to the A/J progenitor strain and the remaining 2 were intergenic deletions unique to NOD/ShiLtJ, consistent with the pattern of these two strains on haplotype analysis (Fig. 2D).

Similarly, variant LOD scores, transcripts, and regulatory elements were identified for the Chr 12 C.I. for W1_EP (Fig. S4) and the Chr 3 C.I. for W4_30C (Fig. S5). Within the Chr 12 W1_EP interval, 23 variants were identified within 1.5 LOD units of the top-scoring variant (rs46944281, LOD = 6.00), with 22 of these being intronic or intergenic and 1 (rs51613251, LOD = 4.52) a missense variant in *Zfyve26*. This variant alters the reference amino acid sequence from alanine in progenitor strains C57BL/6 J, 129, A/J, PWK/PhJ, WSB/EiJ and NZO/HlLtJ, to glutamine in CAST/EiJ and NOD/ShiLtJ mice (https://www.informatics.jax.org/mgv/). This is partially consistent with findings of the W1_EP haplotype analysis (Fig. 2E) discussed above. The Ala/Gln amino acid change in *Zfyve26* is predicted to be structurally tolerated (https://useast.ensembl.org/info/docs/tools/vep/index.html).

Within the Chr 3 C.I. for W4_30C, 250 variants were identified within 1.5 LOD units of the top scoring SNP (rs49087152, LOD = 6.13). Again, most variants occurred in extragenic or intronic sequences; however, there was a missense variant (rs29605696, LOD = 5.10) for *Celsr2*, and 5’-UTR variants for both *Prpf38b* (rs29689050, LOD = 5.59) and *Sypl2* (rs253441023, LOD = 4.69) (https://www.informatics.jax.org/mgv/). The missense variant in *Celsr2* alters a leucine in the reference sequence (i.e., C57BL/6 J, A/J, 129S1/SvImJ, NOD/ShiLtJ, WSB/EiJ) to proline in the NZO/HILtJ, CAST/EiJ, and PWK/PhJ progenitor strains [36]. Haplotype analysis of W4_30C noted above was consistent with this variant strain distribution pattern, demonstrating a decrease in W4_30C for strains containing the proline substitution (Fig. 2I). The Leu/Pro amino acid change in *Celsr2* is predicted to be structurally “tolerated” (https://useast.ensembl.org/info/docs/tools/vep/index.html). The 5’-UTR variant in *Sypl2* is present in NZO/HILtJ, CAST/EiJ, PWK/PhJ, and WSB/EiJ founder strains and falls within a predicted promotor element (ENCODE EM10E0741064). The 5’-UTR variant in *Prpf38b* occurs only in PWK and NZO strains and falls within a predicted binding site for the *Znf549* transcription factor. The strain distribution pattern of the 5’-UTR variants in both *Sypl2* and *Prpf38b* do not fully match the haplotype pattern for the W4_30C phenotype (Fig. 2F), suggesting the rs29605696 missense variant in *Celsr2* as a more likely causal variant for W4_30C.

### eQTL analysis

To identify genetic variance in gene expression within significant bQTL S.I., cis-eQTLs were mapped using RNA-seq data from mPFC. These eQTL and transcriptomic studies are published in detail elsewhere [34] and are described in Supplemental Methods. This analysis identified suggestive (*p* < 0.63, LOD > 6.13) and significant *cis*-eQTLs (*p* < 0.05, LOD > 8.45). When filtered to identify *cis*-eQTLs overlapping the significant W4_EC, W1_EP and W4_30C bQTL confidence intervals, this produced 53 cis-eQTL for 48 genes in these 3 bQTL intervals based on suggestive or significant cis-eQTL, with 45/48 genes having significant (*p* < 0.05) cis-eQTLs (Table 2).

**Table 2:** Top candidate genes from significant bQTL 95% Bayesian confidence intervals.

bQTL	Gene Symbol	Spearman Correlation with Phenotype	Correlation *p* value	Exon Sequence Variants in Top Variants	*cis-*eQTL LOD Score	*cis-*eQTL C.I. (Mbp)	GWAS Associations	Relevant Alcohol/Substance Use Data Set
Week Four Mean 30% Choice (W4_30C; Chr 3)	Wdr77	-0.049	0.4907		15.80	105.83–107.20		[57–59]
	Gstm5	0.125	0.0798		63.64	107.62–108.23	Alcohol drinking [60], alcohol dependence [61]	[62–64]
					44.33	105.95–106.28		
	Gstm7	−0.059	0.4125		113.34	107.66–108.23		
	Pifo	0.027	0.7038		12.89	105.19–107.14		
	Csf1	−0.003	0.9624		19.51	107.65–108.56		[65–67]
	Strip1	0.0124	0.8627		9.26	107.58–108.32		
	Prpf38b	0.008	0.9062	5’-UTR rs29689050 LOD = 5.59	6.57	108.24–109.66		[66, 68]
	Stxbp3	−0.095	0.1822		14.48	107.61–109.19		[64, 68]
	Clcc1	−0.020	0.7822		9.35	107.57–108.73		[66, 68]
	1700013F07Rik	−0.015	0.8356		9.54	107.67–109.19		[69]
	Sypl2	0.107	0.1342	5’-UTR rs253441023 LOD = 4.69	39.11	107.66–108.23		[66, 70]
	Ampd2	0.077	0.2827		7.80	107.55–108.72		[66, 71, 72]
	Gstm4	−0.091	0.2043		45.56	107.61–108.23		[64, 71, 72]
	Kcnc4	0.038	0.5875		12.25	107.43–108.98		[70,72–74]
	Dennd2d	0.233	0.961e-4		5.69	107.61–108.76		[70]
	Vav3	−0.038	0.6000		13.29	108.99–109.66		[66]
	Slc25a24	−0.029	0.6892		17.43	108.99–109.43		
	Fam102b	−0.045	0.5301		11.03	108.51–109.49		[30, 64]
	Elapor1	0.036	0.6135		28.65	108.32–108.71		
	Gstm2	0.060	0.4045		18.68	107.61–108.73		[64,70,72]
	Fndc7	−0.064	0.3727		6.33	108.34–109.69		[75, 76]
	Henmt1	−0.036	0.6161		17.60	108.73–108.98		[66, 69]
	Atxn7l2	0.084	0.2400		17.49	107.62–108.23		[66]
	Ubl4b	0.073	0.3093		14.43	107.55–107.65	Age of onset, alcohol dependence [77]	[70]
	Lrif1	−0.091	0.2041		26.27	105.95–107.14		[66, 78]
	Ntng1	−0.170	0.0166		14.41	109.85–110.95		[79, 80]
	Chia1	0.103	0.1490		61.39	105.95–107.20		[81]
	Tmem167b	−0.117	0.1002		28.14	108.24–108.98		[30, 69, 74]
	Psrc1	0.058	0.4203		10.85	107.63–109.00		[64]
	Mybphl	−0.137	0.0549		23.59	108.24–109.50		
	Gm12522	0.070	0.3276		27.79	108.24–108.97		
	Ovgp1	0.163	0.0225		79.62	105.95–107.14		[69, 74]
	Gm12525	−0.122	0.0871		38.02	107.63–108.51		
	4933431E20Rik	0.150	0.0350		105.29	107.69–108.23		[81]
	Lamtor5	0.033	0.6503		12.40	107.20–108.51		[30, 82, 83]
	Gm37859	0.056	0.4345		10.06	107.63–108.72		
	Gm19391	−0.039	0.5884		15.34	105.95–107.21		
	Gm43745	0.073	0.3107		39.89	109.02–109.50		
	Gm42890	0.119	0.0992		31.53	107.66–108.23		
	Adora3	−0.076	0.2888		62.18	105.95–107.20		[64]
	Eps8l3	NA	NA		NA		Alcohol drinking [60], alcohol dependence [61]	
	Celsr2	0.103	0.1503	Missense rs29605696 LOD = 5.10	NA		Cholesterol x alcohol consumption interaction [49], Forns index in high alcohol intake [48]	[30, 59, 84]
Week Four Mean Ethanol Consumption (W4_EC; Chr 4)	Car8	−0.221	0.0069		27.59	7.60–8.15		[59, 69, 72, 79, 85]
Week One Mean Ethanol Preference (W1_EP; Chr 12)	Mpp5/Pals1	−0.212	0.0189		9.62	76.97–78.93		[70, 81]
	Atp6v1d	0.039	0.7597		19.08	79.86–80.03		[64, 69, 75, 86]
					17.41	80.09–80.56		
	Rdh12	−0.053	0.6622		9.02	79.21–80.14		[66,87–89]
	Vti1b	−0.062	0.6020		11.98	77.76–79.75		[58, 66, 80, 90]
					13.57	79.91–80.14		
	Arg2	−0.049	0.6897		23.64	78.18–79.33		[65]
	Tmem229b	0.046	0.7168		18.72	79.22–79.60		[69, 80, 81]
	Zfyve26	0.0004	0.9983	Missense rs51613251 LOD = 4.52	41.29	78.09–79.60		[66, 70]
					27.14	80.17–80.47		
	Rdh11	0.009	0.9543		29.85	78.09–79.19		[64, 66, 87, 88]
					21.23	80.14–80.47		

Top candidates having either a cis-eQTL (LOD > 6.13) from DO mouse PFC or an association (*p* < 5.0 × 10E-6) with human alcohol-related traits from GWAS Catalog. Genes are presented within each QTL in order of chromosomal location using starting position in Mbp.

Only one gene within the significant bQTL confidence interval for W4_EC on Chr 4 had a *cis*-eQTL which met filtering criteria: *Carbonic anhydrase 8* (*Car8*) (Fig. 3B). Haplotype analysis of the *Car8 cis-*eQTL indicated that A/J alleles at the locus correlated with increased expression of *Car8* in prefrontal cortex (Fig. 3C). *Car8* expression had a significant negative Spearman correlation with both W4_EC (*r* = −0.22; *p* = 0.008) and week four ethanol preference (*r* = −0.23; *p* = 0.006), consistent with the relationship suggested by the effect of A/J alleles. Taken together, these results suggest a role for variants unique to A/J mice in both decreased *Car8* expression and increased last week ethanol consumption in DO mice.

The significant Chr 3 bQTL for W4_30C contained 40 genes having cis-eQTLs with LOD scores > 6.13 (Table 2). Of these, *Sypl2* and *Prpf38b* also contained 5’-UTR sequence variation as noted above. No *cis*-eQTL was observed for *Celsr2* at this locus. Eight genes within the Chr 12 W1_EP bQTL C.I had significant cis-eQTLs with LOD scores > 8.45 and four of these having multiple significant eQTLs. *Zfyve26* had two significant eQTLs and a coding sequence variant in the top variants as noted above.

### Bioinformatics analysis identifies genes with alcohol trait human GWAS associations

Bioinformatics analysis identified 7 genes from significant or suggestive bQTLs support intervals as having significant or suggestive associations (*p* values < 6.0E-6) with alcohol-related traits in the GWAS Catalog database (https://www.ebi.ac.uk/gwas/home) and multiple genes with supporting literature associations with ethanol or substance misuse (Table 2). Of the significant bQTLs, 4 human GWAS hits occurred within the LW30C Chr 3 S.I. *Gstm5* is related to glutathione metabolism, *Eps8l3 and Celsr2* function in epidermal growth factor pathways, and *Ubl4b* codes for a ubiquitin-like protein functioning in protein targeting.

## Discussion

This report is the first to analyze genetic variance in ethanol consumption using DO mice. The design of this work allowed study of a progressive ethanol consumption phenotype at high genetic resolution in a model assessing a large proportion of genetic variance across mouse populations. Our results show remarkable diversity in ethanol consumption across the study population and a suggestion of differing genetic influences on initial ethanol intake (week one) versus chronic consumption (week four). We identified 3 significant and 13 suggestive bQTLs across 7 chromosomes. In most cases, the support intervals for these bQTL in DO mice were much narrower (Table 2) than seen in prior mouse genetic studies on ethanol behaviors. The integration of cis-eQTL and bioinformatic analyses allowed prioritization of an experimentally tractable number of novel high priority candidate genes, with some candidates overlapping results from prior rodent or human GWAS studies on alcohol.

### bQTL analysis identifies specific, novel loci for ethanol consumption behaviors

Our high-resolution genetic mapping for ethanol consumption phenotypes identified significant or suggestive bQTLs across assays in week one or week four of ethanol access (Table 1), but importantly, we did not observe any bQTLs in common between those time periods, suggesting differing genetic influences on initial ethanol consumption and more chronic, progressive consumption. This is also consistent with our multivariate analysis of the longitudinal consumption data, showing a clear separation in initial vs. late ethanol access. Only one suggestive bQTL was identified for week 4–week 1 consumption, on Chr 15 in a region overlapping with a suggestive QTL for W4_EP, despite the strong evidence for significant escalation of ethanol consumption in the overall study. This likely resulted from decreased statistical power due to the mathematical manipulation in the difference score.

Several of the mapped bQTLs overlapped regions implicated in prior rodent genetic model analysis of ethanol or substance use behaviors (Table 1). For example, all 3 bQTLs on Chr 3 were located within the confidence interval for alcohol preference identified by Belknap and Atkins in 2001 [11, 37]. However, the QTL confidence intervals identified here were generally an order of magnitude smaller than prior reports, with the largest being the significant interval for W4_30C at approximately 3.77 Mbp. These comparisons with prior studies demonstrate both the power of our DO mouse analysis for high resolution identification of novel loci modulating ethanol consumption, and the likely validation and improved mapping of prior rodent QTL analyses, consistent with other behavioral genetic studies using DO mice [28, 38].

### eQTL and bioinformatics analysis identifies Car8 as a top candidate gene for Chr4 LWEC QTL

We identified *cis-eQTL* within bQTL support intervals to annotate candidate gene lists since such colocalization provides strong evidence for genetic modulation of gene expression influencing ethanol-related behaviors. A prime example of this approach was *Car8*, located within the significant Chr 4 bQTL for W4_EC (Fig. 3). *Car8* was the only gene in the interval having a significant cis-eQTL in our mPFC data. Furthermore, haplotype analysis across the W4_EC bQTL and Car8 eQTL revealed that A/J alleles at this locus correlated with both decreased week 4 ethanol consumption and increased *Car8* expression in prefrontal cortex. *Car8* expression in mPFC notably had a significant negative correlation with ethanol consumption in our male Diversity Outbred mice. We consider these data strong support for *Car8* as a high priority candidate gene in the Chr4 W4_EC bQTL.

*Car8* shares sequence similarity to the carbonic anhydrase family of genes, but lacks carbonic anhydrase activity; instead, it is known to inhibit the IP3R1 calcium signaling channel [39]. IP3R1 has been suggested to play a role in ethanol-enhanced GABA release in cerebellum, a potential mechanism by which ethanol-induced cerebellar ataxia occurs [40]. *Car8* is expressed across multiple brain regions and implicated in locomotor function and analgesia [41–43]. *Car8* has been shown to be regulated by ethanol within cerebellum, ventral midbrain and anterior cingulate in mice (Table 2). Additionally, recent reports have suggested that IP3R1 signaling in prefrontal cortex astrocytes can regulate ethanol consumption and that *Car8* is regulated in astrocytes within that brain region by chronic ethanol consumption [20, 44]. Given this ethanol regulation in brain, the significant negative correlations between *Car8* expression and W4_EC (Fig. S6), and the strong *Car8* eQTL in linkage disequilibrium with the Chr 4 bQTL for W4_EC, we hypothesize that altered expression of *Car8* may modulate chronic ethanol consumption.

### Other bQTL candidates with structural variants or links to human GWAS

Several candidate genes within the significant bQTL for W4_30C on Chr 3 (*Celsr2, Prpf38b* and *Sypl2*) and W1_EP on Chr 12 (*Zfyve26*) contained exonic sequence variants (Table 2) that might modulate gene expression or function. Of note, *Prpf38b* is poorly characterized functionally but is regulated by ethanol in prefrontal cortex astrocytes, as noted above for *Car8* [45]. *Celsr2* encodes a transmembrane protein and has been implicated in axon development in the forebrain [46] and regulation of motor neuron regeneration following injury [47]. Notably, *Celsr2* had a missense variant in the DO population and GWAS data show that *Celsr2* is strongly associated with liver fibrosis in individuals with high alcohol intake [48] and with interaction terms between alcohol consumption and both LDL and HDL cholesterol levels [49]. Additionally, *Celsr2* has previously been shown to be regulated by ethanol in chronic ethanol consuming rhesus macaques [50] and in multiple rodent studies [30, 45, 51]. These genes with exonic variants clearly warrant further study to characterize any direct involvement in ethanol-related behaviors. Overall, the consilience between studies here, prior model organism behavioral genetics and human GWAS studies serve to reinforce our findings and justify pursuit of further validation or mechanistic studies in mice.

### Limitations

Despite the novel findings of this analysis, there are several limitations which should be considered. Firstly, the Diversity Outbred mouse population used for this study only included male animals due to power considerations; while future studies are planned to validate identified loci and candidate genes in female mice, these results may not be generalizable to both sexes. Additionally, RNA-seq data used for eQTL analysis came from bulk sequencing of only prefrontal cortex tissue, which may not reflect transcriptomic differences in individual cell types or other brain regions. Larger sample sizes, incorporation of expression data from additional brain regions, and a single cell RNA-seq approach would provide more detailed insight into the genetic and transcriptomics mechanisms underlying observed differences in ethanol consumption. Finally, the addition of human GWAS data as an additional criterion for identifying top candidate genes is limited by the methods in which GWAS associations are linked to individual genes and the size of existing studies.

## Conclusions

These findings identified novel genes and potential mechanisms modulating ethanol consumption phenotypes in DO mice, thus adding to our understanding of the complex genetic and molecular architecture of ethanol consumption and AUD in humans. Further transcriptome, bioinformatic and behavioral genetic analyses of this powerful dataset promise to identify additional gene networks or individual targets that may aid in development of future therapeutic approaches for AUD [34].

## Supplementary information

Supplemental Materials

## Data Availability

All data supporting the conclusions of this manuscript are contained in figures and tables or supplemental materials. Cleaned behavioral and genotype data and expression QTL data are provided on the Open Science Framework (https://osf.io/tfrzg/?view_only=232cfcc8d9a94d16846138f64e4e3e59). Additional raw data will be provided upon request.
