# Supplementary material for: Identification of novel genetic loci and candidate genes for progressive ethanol consumption in diversity outbred mice
Source: Neuropsychopharmacology. 2024 Jun 29;49(12):1892–904. doi: 10.1038/s41386-024-01902-6 (PMC11473901; doi:10.1038/s41386-024-01902-6)
Supplement: Supplementary file 1 — Supplemental Materials [file 41386_2024_1902_MOESM1_ESM.pdf]

Supplemental Methods

Ethics Statement

All animal care and euthanasia procedures were performed in accordance with the rules and regulations established by the United States Department of Agriculture Animal Welfare Act and Regulations, Public Health Services Policy on Humane Care and Use of Laboratory Animals, and American Association for Accreditation of Laboratory Animal Care. Humane endpoints were established by the same standards.

Animal Housing and Power

Male DO mice (n = 636) were acquired from Jackson Laboratories after weaning at 4-6 weeks of age in 7 cohorts (average n = 106) spanning DO generations 22-25. Mice were singly-housed in temperature- and humidity-controlled vivariums on cedar shaving bedding with *ad libitum* access to water and standard chow (#7912, Harlan Teklad, Madison, WI, United States). Vivariums were set up with alternating 12-hour light and dark phases, and all mice were weighed weekly. Experimental procedures were begun on animals at 8-12 weeks of age.

The number of animals utilized in this work was planned based on prior studies that estimated ~600 DO mice were required to achieve 80% power to detect alleles causing less than 5% variance in a trait [1]. Female mice were excluded in this experiment because of likely sex-driven differences in ethanol consumption, including known differences in male and female ethanol consumption patterns in one of the DO progenitor strains, C57BL/6J. Including both sexes in this study would have required an unworkable number of animals (n>1200) to achieve adequate genetic power.

Intermittent Ethanol Access

Mice (n = 587) were exposed to ethanol via a three-bottle choice IEA paradigm for 5 weeks. (Figure 1A) This consisted of alternating 24-hour periods of ethanol access (Monday, Wednesday, Friday), during which time mice were presented with three bottles (15% ethanol v/v, 30% ethanol v/v, and water). Ethanol was presented at the beginning of the dark phase and consumption was measured in mL 24 h later at the end of the following light phase. Bottles of each fluid were also placed on empty cages to correct for evaporation and spillage, and placement order of bottles was randomized each IEA day to avoid place preference confounds. Ethanol and water volume measurements were used to derive dependent variable values for ethanol consumption (g ethanol/kg body weight per 24 hours), ethanol preference compared to water (total volume

ethanol consumed/total fluid consumed) and for volume of 30% ethanol compared to volume of 15% ethanol ("30% choice"). 49 animals were exposed to only water as controls for anxiety-related behavioral studies and RNAseq analyses, with consumption measured in mL on the treatment group's IEA days. Daily drinking values were analyzed to identify outliers, leading to one daily value for one sample being removed.

Although not included in this report, all DO animals also underwent locomotor and anxiety-like behavioral testing via a light-dark box assay [2] 24 hours prior to initiation of IEA. Furthermore, after 4 weeks of IEA, animals were tested for anxiety-like behavior using a marble-burying assay at 24 hours after last access to ethanol. IEA consumption was then continued for 1 week (3 drinking days) prior to tissue harvesting as below . Only the first 4 uninterrupted weeks of IEA were used for the purposes of behavioral genetic studies in this report, excluding day 1 of IEA due to increased variance on initial exposure to ethanol bottles.

Analysis of Behavioral Data

To identify the presence of any patterns in ethanol consumption, principal component analysis was conducted using daily ethanol consumption data in grams of ethanol per kilogram of body weight. This identified several major principal components loading differently on the ethanol consumption time course (Supplemental Figure 3). The top 2 principal components were tested as additional phenotypes in genetic mapping (Supplemental Figure 3). Hierarchical clustering was carried out using Ward's method on daily ethanol consumption data (in grams of ethanol per kilogram of body weight)[3]. Because of the presence of two superclusters which roughly divided drinking days into early and late phases, mean ethanol consumption, preference, and 30% choice phenotypes were derived for week 1 (drinking days 2-4), week 4 (drinking days 9-11), and whole study time periods (drinking days 2-11). Additionally, a week 4 -week 1 difference in total ethanol consumption was calculated as an additional phenotype (Supplemental Figure 7). Linear modeling of daily consumption and within-sample t-tests were used to compare week 1 and week 4 consumption and characterize progressive ethanol consumption across our mouse population.

Tissue Sample Collection and Genotyping

Mice were sacrificed 24 hours after the end of their last ethanol exposure period via cervical dislocation and decaptitation and tissue samples were collected immediately afterwards, flash-frozen in liquid nitrogen and stored at -80° C. Tail snips were collected and sent to NeoGen Inc (Lincoln, NE) for DNA isolation and genotyping using a GigaMUGA microarray (n_{SNP} = 141,090; n_{CNV} = 2,169) designed to optimize genotyping of

DO mice.[4] Mice were genotyped in two batches ($n_1 = 240$; $n_2 = 420$). Brains were microdissected into nine regions as previously described [5, 6]. Samples were only collected from 630 mice as 6 mice reached humane endpoints before the end of experimentation. After genotyping, 21 mice were removed due to low overall call quality (< 98% call rate), and one additional mouse was removed due to high heterozygosity (> 3 standard deviations above the mean) indicating contamination. Seven additional mice were removed due to potential sample mix-ups during data collection, resulting in a final sample of 603 mice (554 ethanol-drinking mice and 49 ethanol-naïve controls).

Behavioral QTL Analysis

QTL mapping was carried out using the R/QTL2 software package for R. Dependent variables included whole study, week 1, and week 4 averages for ethanol consumption, ethanol preference, and 30% choice [7]. SNPs were imputed based on progenitor haplotype probabilities at intervals of 0.01 Mbp and relatedness between individual mice was controlled for using a kinship matrix derived using the leave-one-chromosome-out (LOCO) method [8]. Cohort was included as a fixed-effect covariate in all of our analyses. Phenotypes were either log- (for consumption and 30% choice) or square-root-transformed (for ethanol preference) to obtain normality before running analyses. Genome-wide significance thresholds were derived using permutation analysis ($n_{perm} = 1000$), with an empirical threshold of $p < 0.05$ used to identify significant loci and a conventionally-used threshold of $p < 0.63$ used to identify suggestive loci. 95% Bayesian confidence intervals were identified for both significant and suggestive QTLs and applied to bQTL, haplotype, and SNP analyses.

Positional candidate genes were identified as genes with coding regions located within significant bQTL confidence intervals using gene annotation databases from Mouse Genome Informatics (MGI).[9] To detect founder strain effects, haplotype analysis was conducted for chromosomes containing significant or suggestive bQTLs using best linear unbiased predictors (BLUPs) in R/QTL2. Lastly, LOD scores for individual SNP variants within significant bQTL confidence intervals were estimated using R/QTL2. Significance levels for SNP variants were determined by permutation. The OrganismDbi package in R [10], together with information from MGI and ReMap [11], were then used to annotate known gene transcripts and regulatory elements from ChIP-seq, respectively, aligned to the GRCm38/mm10 genome assembly.

RNA Extraction and Sequencing

Transcriptomics and eQTL analysis is described more fully in an additional preprint manuscript from our laboratory[12]. Prefrontal cortex samples from 220 mice were chosen for RNA-seq analysis based on average total ethanol consumption during the fourth week of IEA. This included 100 mice from each extreme of the ethanol consumption distribution and an additional 20 ethanol-naïve control mice. RNA was extracted and purified using RNeasy mRNA kits (Qiagen, Germantown, MD). Quality of isolated RNA was assessed using both a NanoDrop assay (Thermo Fisher Scientific, Waltham, MA) and BioAnalyzer 2100 (Agilent Technologies, Santa Clara, CA). Isolated RNA samples were then shipped on dry ice for poly-A selected library construction and sequencing at the Novogene Bioinformatics Institute (Sacramento, CA) as paired-end sequence 150 bp fragments with approximately 20 million reads per sample on the Illumina HiSeqX system.

RNA-seq quality control was performed using FastQC 0.11.7 and based on several metrics including base sequence quality, tile sequence quality, base sequence content, sequence length, duplication levels, and adapter content [13]. Trimmomatic 0.38 was used to trim the first 5 bp from the 3` end of each read and to trim the 5` end based on read quality [14]. After passing QC, a pooled transcriptome of all DO mouse founder strains was used to reconstruct and align to individual genomes for each subject using Genotype-by-RNA-Seq (GBRS)[15]. Finally, variant-stabilized transcript counts (VST) were quantified using DESeq2 [16].

Expression QTL Analysis

eQTL mapping was carried out using R/QTL2 software. VST-normalized GBRS expression data for individual gene transcripts were inserted as phenotypes, with the LOCO-derived kinship matrix and fixed-effect cohort covariates included in the analyses. Due to the large number of expression QTL (eQTL) analyses conducted, permutation analysis was computationally impractical for all eQTLs mapped. We therefore performed permutation analysis (n=1000) on a random subset of genes (n=220) representing approximately 1% of the total transcriptome. This produced suggestive significance ($p<0.63$, LOD≥6.13) and significant ($p<0.05$, LOD≥8.45) thresholds for filtering eQTL. eQTLs were further filtered to identify *cis*-eQTLs located < 2 Mbp from the correlated gene's start or stop position and with 95% Bayesian confidence intervals < 2 Mbp in size. *D`* linkage disequilibrium estimates were then calculated between markers within confidence intervals of *cis*-eQTLs corresponding to positional candidate genes and peak bQTL markers [17].

Bioinformatics Analysis of Candidate Genes

Variants including single nucleotide polymorphisms (SNPs), copy number variants/microsatellite regions, and insertions/deletions within significant bQTL confidence intervals were identified using the dbSNP annotation database from MGI [18]. dbSNP provides information on each variant's predicted function class, defined by positional relatedness to a specific transcript for a gene. Variants within coding sequences and which change amino acid sequence, which are more likely to cause functional consequences to positional candidate genes, were identified using this database as well as variants with founder strain distributions matching observed haplotype patterns.

GWAS Catalog was searched for associations between human orthologs of positional candidate genes and alcohol-related traits [19]. Positional candidate genes were also used as search terms in GeneWeaver (http://geneweaver.org) to identify data sets related to ethanol or other substance use [20]. Finally, positional candidate genes were compared with significance GWAS results from a recent large meta-analysis for problematic alcohol consumption (PAU) [21].

The GeneNetwork web resource (https://genenetwork.org) was used to detect whether *Car8* expression was also correlated with ethanol consumption in the BXD recombinant inbred panel derived from C57BL/6J and DBA/2J mice, as seen in DO mice. This analysis was initially conducted using PFC expression datasets and although very strong correlations were identified, there was only a limited number of BXD lines in common between the two data types for that brain region. Inquiry in GeneNetwork for other brain region datasets identified a *Car8* expression QTL on Chromosome 4 in Cerebellum with a strong cis-eQTL (peak -logP = 30.64) and large number of samples (n=57) available in that tissue dataset (GeneNetwork dataset: GenEx BXD Sal Cerebellum Affy M430 2.0 (Feb13) RMA both sexes; probeset 1457904_at). Spearman correlation of this expression dataset with GeneNetwork BXD Published Phenotypes database identified multiple significant correlations to datasets from a recently published drinking-in-the-dark 20% ethanol binge ethanol consumption study [22]. A control baseline 20% ethanol consumption in females over 2h drinking phenotype from that study (GeneNetwork trait BXD_20188) was therefore used for Spearman correlation analysis with cerebellar *Car8* expression (Suppl. Figure 6) using the stats package for R. There were 34 BXD strains were in common between the drinking-in-the-dark phenotype and cerebellar *Car8* expression. Genotypes of strains were determined at a peak marker for Car8 expression (rs32484153).

References for Supplemental Methods

1. Gatti, D.M., et al., *Quantitative trait locus mapping methods for diversity outbred mice.* G3 (Bethesda), 2014. **4**(9): p. 1623-33.
2. Putman, A.H., et al., *Identification of quantitative trait loci and candidate genes for an anxiolytic-like response to ethanol in BXD recombinant inbred strains.* Genes Brain Behav, 2016. **15**(4): p. 367-81.
3. Ward, J.H., *Hierarchical Grouping to Optimize an Objective Function.* Journal of the American Statistical Association, 1963. **58**(301): p. 236-244.
4. Morgan, A.P., et al., *The Mouse Universal Genotyping Array: From Substrains to Subspecies.* G3 (Bethesda), 2015. **6**(2): p. 263-79.
5. Wolen, A.R., et al., *Genetic dissection of acute ethanol responsive gene networks in prefrontal cortex: functional and mechanistic implications.* PLoS One, 2012. **7**(4): p. e33575.
6. Kerns, R.T., et al., *Ethanol-responsive brain region expression networks: implications for behavioral responses to acute ethanol in DBA/2J versus C57BL/6J mice.* J Neurosci, 2005. **25**(9): p. 2255-66.
7. Broman, K.W., et al., *R/qtl2: Software for Mapping Quantitative Trait Loci with High-Dimensional Data and Multiparent Populations.* Genetics, 2019. **211**(2): p. 495-502.
8. Cheng, R., et al., *Practical considerations regarding the use of genotype and pedigree data to model relatedness in the context of genome-wide association studies.* G3 (Bethesda), 2013. **3**(10): p. 1861-7.
9. Bult, C.J., et al., *Mouse Genome Database (MGD) 2019.* Nucleic Acids Res, 2019. **47**(D1): p. D801-D806.
10. Carlson M, P.H., Morgan M, Obenchain V, *OrganismDbi: Software to enable the smooth interfacing of different database packages.* 2022. p. R package version 1.38.1.
11. Hammal, F., et al., *ReMap 2022: a database of Human, Mouse, Drosophila and Arabidopsis regulatory regions from an integrative analysis of DNA-binding sequencing experiments.* Nucleic Acids Res, 2022. **50**(D1): p. D316-D325.
12. Smith, M.L., et al., *Identification of Genetic and Genomic Influences on Progressive Ethanol Consumption in Diversity Outbred Mice.* bioRxiv, 2023.
13. Andrews, S., *FastQC: A quality control tool for high throughput sequence data*. 2015.
14. Bolger, A.M., M. Lohse, and B. Usadel, *Trimmomatic: a flexible trimmer for Illumina sequence data.* Bioinformatics, 2014. **30**(15): p. 2114-20.
15. Choi, K., et al., *Genotype-free individual genome reconstruction of Multiparental Population Models by RNA sequencing data*. 2020, Cold Spring Harbor Laboratory.
16. Love, M.I., W. Huber, and S. Anders, *Moderated estimation of fold change and dispersion for RNA-seq data with DESeq2.* Genome Biol, 2014. **15**(12): p. 550.
17. Lewontin, R.C., *The Interaction of Selection and Linkage. I. General Considerations; Heterotic Models.* Genetics, 1964. **49**(1): p. 49-67.
18. Blake, J.A., et al., *Mouse Genome Database (MGD): Knowledgebase for mouse-human comparative biology.* Nucleic Acids Res, 2021. **49**(D1): p. D981-D987.
19. Buniello, A., et al., *The NHGRI-EBI GWAS Catalog of published genome-wide association studies, targeted arrays and summary statistics 2019.* Nucleic Acids Res, 2019. **47**(D1): p. D1005-D1012.
20. Baker, E.J., et al., *GeneWeaver: a web-based system for integrative functional genomics.* Nucleic Acids Res, 2012. **40**(Database issue): p. D1067-76.
21. Zhou, H., et al., *Multi-ancestry study of the genetics of problematic alcohol use in over 1 million individuals.* Nat Med, 2023. **29**(12): p. 3184-3192.
22. Mulligan, M.K., et al., *Impact of Genetic Variation on Stress-Related Ethanol Consumption.* Alcohol Clin Exp Res, 2019. **43**(7): p. 1391-1402.

Supplemental Table 1. Ethanol consumption phenotypes are heritable in our DO mouse population. Heritability estimates were estimated using restricted maximum likelihood in R/qtl2.

Trait	Time Period	h^2
Ethanol Consumption	Whole Study	0.299
	Week One	0.197
	Week Four	0.31
Ethanol Preference	Whole Study	0.267
	Week One	0.143
	Week Four	0.301
30% Choice	Whole Study	0.073
	Week One	0
	Week Four	0.041

Supplemental Table 2. Top SNPs from significant bQTL for week four mean ethanol consumption on Chromosome 4. LOD scores were calculated for individual variants across the 95% Bayesian C.I. for the W4_EC bQTL. Top variants were identified as those within 1.5 LOD units of the top-scoring variant. Empiric p-values thresholds for significance are as on Figure 3A (*p<0.63).

Variant	Type	Position (Mbp)	Alleles (Reference|Variant)	Consequence	SNP LOD	Strain with Variant Allele
4:8302313_A/C	SNP	8.302313	*A|C*	Intergenic variant	3.25*	A/J
4:8384190_G/A	SNP	8.38419	*G|A*	Intergenic variant	3.71*	A/J
rs249655952	SNP	8.437965	*T|A*	Gm37386 downstream variant	3.84*	A/J
SV_4_8917963_8918080	Structural Variant	8.918022	-|*4:8917963-8918080;DEL;RAW;DEL*	NA	2.63	NOD/ShiLtJ
rs32455519	SNP	9.164587	*C|T*	Intergenic variant	3.18*	A/J
rs262840752	SNP	9.241249	*C|T*	Intergenic variant	2.96*	A/J
SV_4_9432463_9432570	Structural Variant	9.432516	-|*4:9432463-9432570;DEL;RAW;DEL*	NA	2.92*	NOD/ShiLtJ

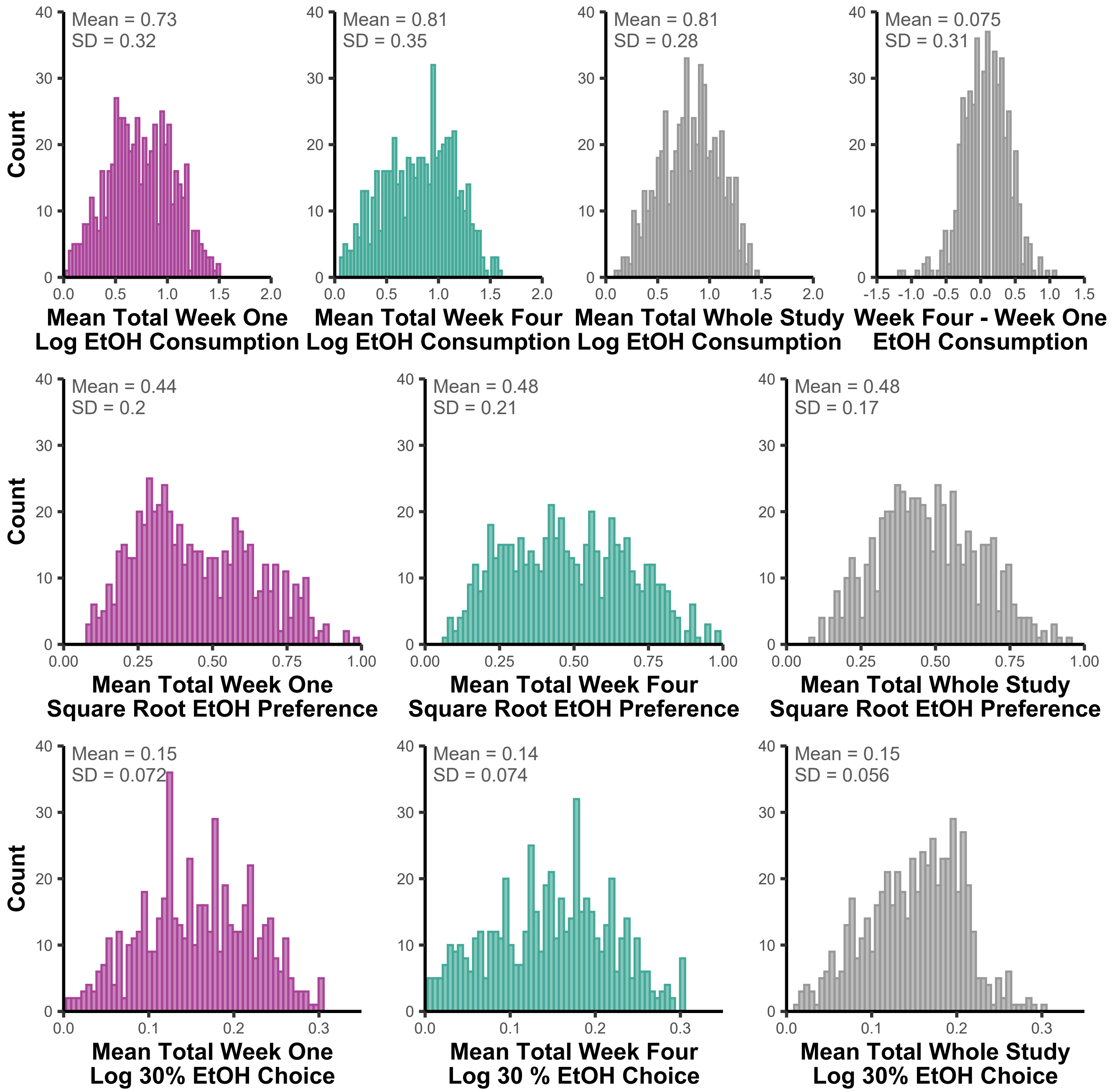

Supplemental Figure 1. Histograms of whole study, week one, and week four ethanol consumption, preference, and 30% choice following normalization show variability in ethanol drinking phenotypes across DO mice. Upper panels: Week one (drinking days 2-4), week four (drinking days 9-11), whole study (drinking days 2-11) and (week four – week one) mean consumption values were calculated for each mouse passing quality control analysis (*n* = 554). Middle panels: Ethanol preference versus over water distributions for week one, week four and total study. Lower panels: Choice for 30% (v/v) ethanol concentration compared to total ethanol ("30% ethanol choice"). Consumption and 30% ethanol choice were log-transformed for normalization; preference was square root-transformed; Week four – week one consumption used transformed values for calculation.

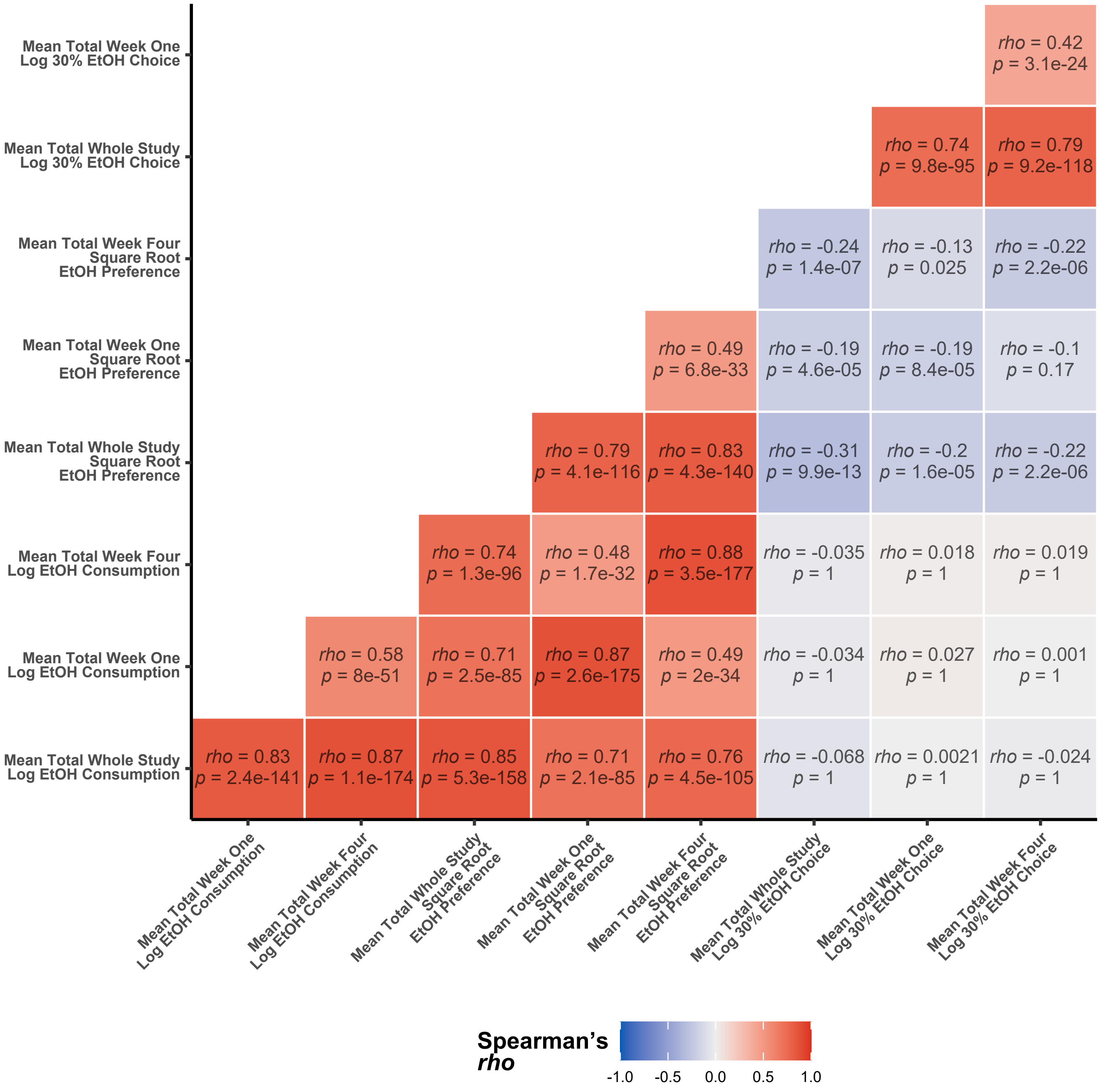

Supplemental Figure 2. Spearman correlations between ethanol consumption behaviors. Ethanol consumption, preference, and 30% choice were measured over 4 weeks of IEA via three-bottle choice. Spearman correlations were calculated for week one and week four phenotypes. Means were significantly positively correlated within each phenotype; preference also significantly positively correlated with consumption for each time period. 30% choice was negatively correlated with preference, but not significantly correlated with overall consumption.

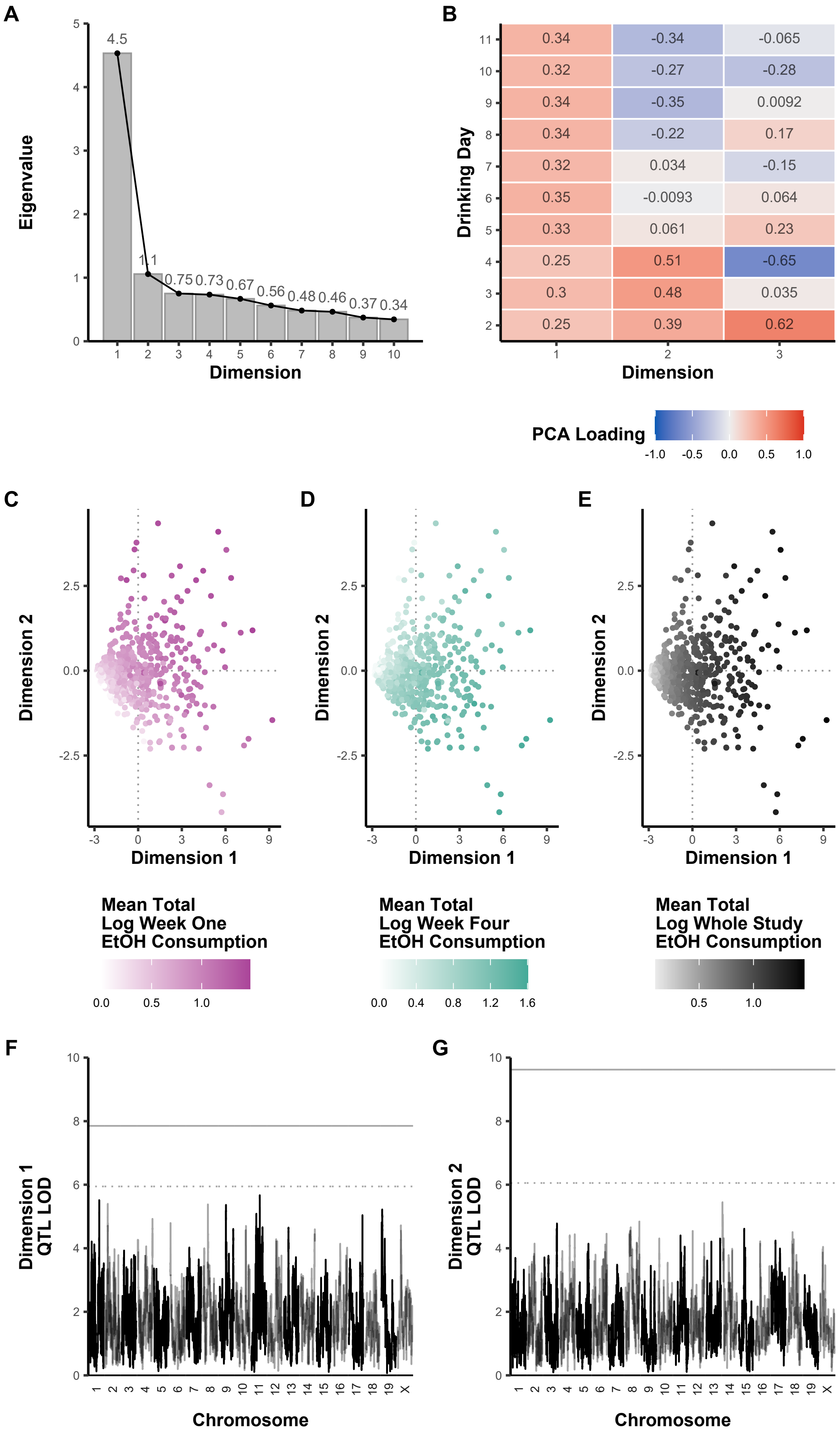

A
Eigenvalue
Dimension
4.5
1.1
0.75 0.73 0.67 0.56 0.48 0.46 0.37 0.34
B
Drinking Day
Dimension
PCA Loading
-1.0 -0.5 0.0 0.5 1.0
C
D
E
Dimension 2
Dimension 1
Mean Total Log Week One EtOH Consumption
Mean Total Log Week Four EtOH Consumption
Mean Total Log Whole Study EtOH Consumption
F
G
Dimension 1 QTL LOD
Dimension 2 QTL LOD
Chromosome

Supplemental Figure 3. Principal component analysis of daily drinking values across DO mice reveals main effect of whole study mean ethanol consumption. A single principal component explained 42.4% of the total variance in daily drinking across 636 male DO mice (A). Component loading across the first component was relatively even across drinking days, whereas the second component was positively loaded by early drinking days (2-4) and negatively loaded by later drinking days (8-11) (B). The first component appears correlated with whole study mean ethanol consumption (C), while principal component 2 appears positively correlated with week one (days 2-4) mean ethanol consumption (D) and negatively correlated with week four (days 9-11) mean ethanol consumption (E). Principal components 1 (F) and 2 (G) were both used as phenotypes for QTL mapping, but no significant (solid line, $p = 0.05$) or suggestive (dashed line, $p = 0.63$) LOD peaks were identified.

A

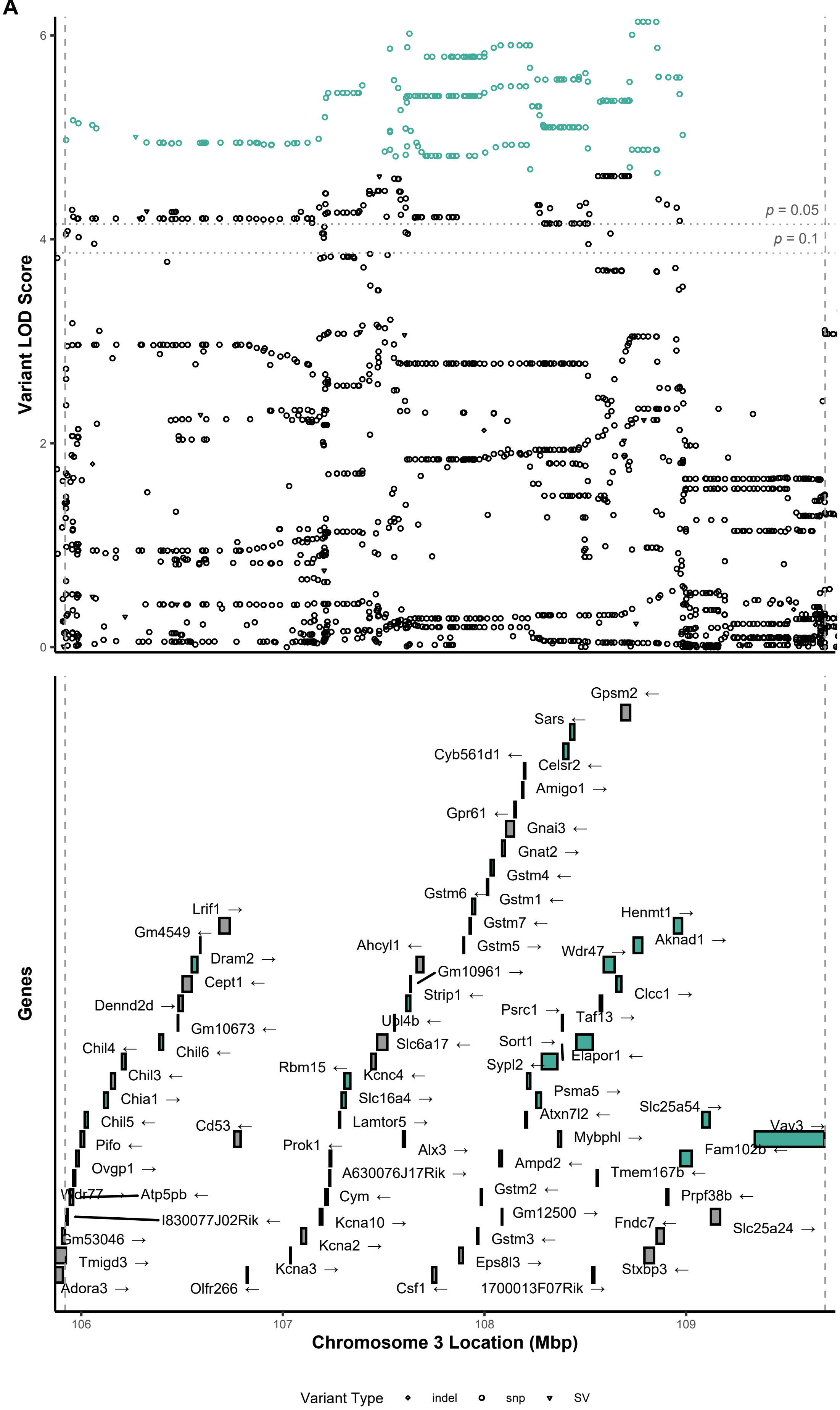

Variant LOD Score
p = 0.05
p = 0.1
Genes
Chromosome 3 Location (Mbp)
106
107
108
109
0
2
4
6
Gpsm2 ←
Sars ←
Cyb561d1 ←
Celsr2 ←
Amigo1 →
Gpr61 ←
Gnai3 ←
Gnat2 →
Gstm4 ←
Gstm6 ←
Gstm1 ←
Lrif1 →
Henmt1 →
Gm4549 ←
Gstm7 ←
Aknad1 →
Ahcyl1 ←
Gstm5 →
Wdr47 →
Dram2 →
Gm10961 →
Cept1 ←
Strip1 ←
Clcc1 →
Dennd2d →
Psrc1 →
Ubl4b ←
Taf13 →
Gm10673 ←
Slc6a17 ←
Sort1 →
Chil4 ←
Chil6 ←
Elapor1 ←
Rbm15 ←
Sypl2 ←
Chil3 ←
Kcnc4 ←
Psma5 →
Chia1 →
Slc16a4 →
Slc25a54 →
Chil5 ←
Lamtor5 →
Atxn7l2 ←
Cd53 ←
Vav3 →
Pifo ←
Prok1 ←
Mybphl →
Alx3 →
Fam102b ←
Ovgp1 →
Ampd2 ←
A630076J17Rik →
Tmem167b ←
Wdr77 →
Atp5pb ←
Gstm2 ←
Cym ←
Prpf38b ←
Gm12500 →
I830077J02Rik ←
Kcna10 →
Fndc7 ←
Slc25a24 →
Gm53046 →
Gstm3 ←
Kcna2 →
Tmigd3 →
Eps8l3 →
Kcna3 →
Stxbp3 ←
Adora3 →
Olfr266 ←
Csf1 ←
1700013F07Rik →
Variant Type
indel
snp
SV

Supplemental Figure 4. Variant LOD scores across significant bQTL on chromosome 12 for week one mean ethanol preference (W1_EP). (A) SNP associations were estimated across the 95% Bayesian confidence interval identified for the significant bQTL, identified by dashed vertical lines. Top variants (green) were identified as those with a LOD score within 1.5 of the highest score. Statistical thresholds identified by permutation analysis are indicate as horizontal dotted lines. (B) Known gene transcript annotations within this C.I. are identified. Prioritized interval genes from Table 2 are highlighted in green. These include *Celsr2* with missense mutations.

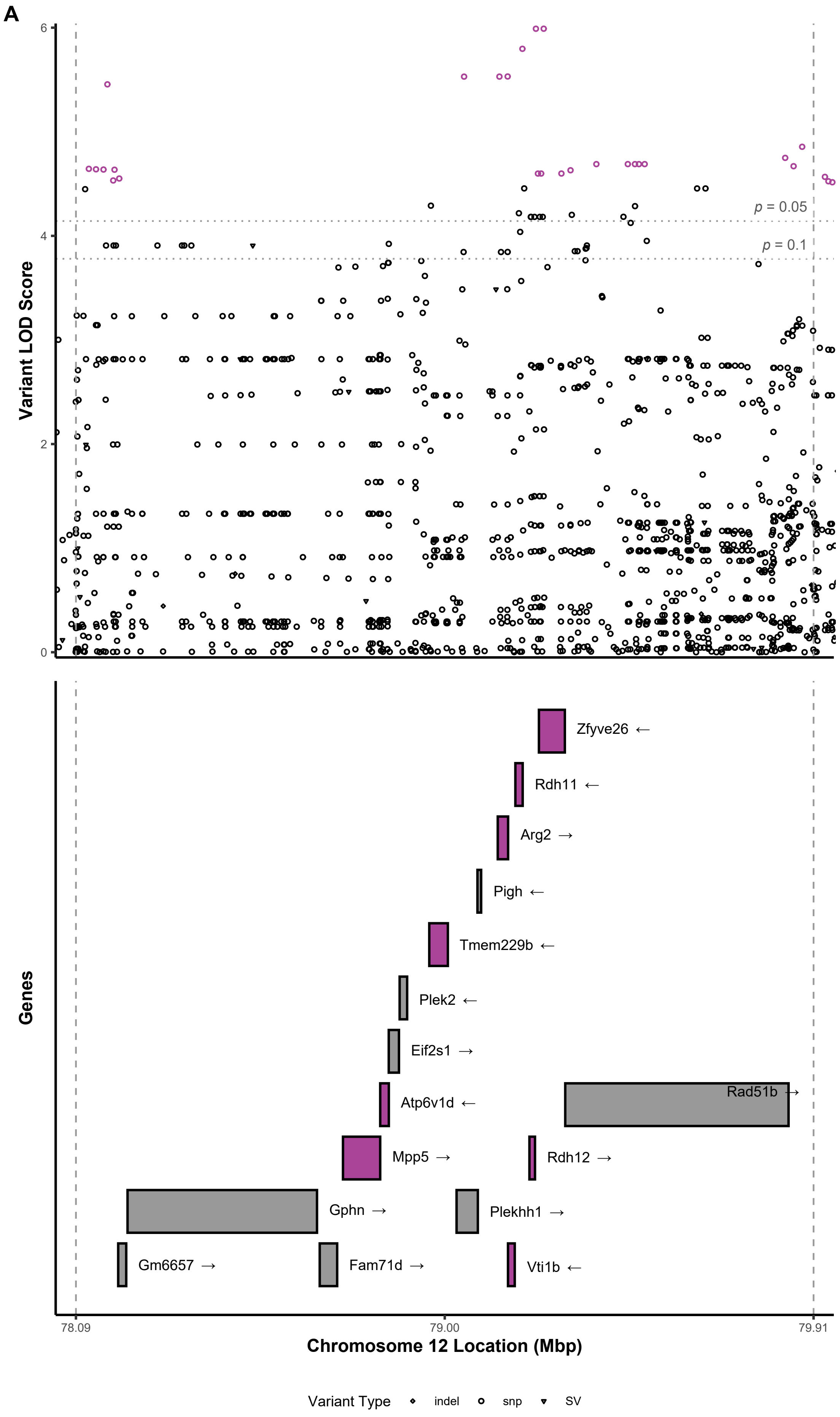
A
Variant LOD Score
p = 0.05
p = 0.1
Genes
Zfyve26 ←
Rdh11 ←
Arg2 →
Pigh ←
Tmem229b ←
Plek2 ←
Eif2s1 →
Atp6v1d ←
Rad51b →
Mpp5 →
Rdh12 →
Gphn →
Plekhh1 →
Gm6657 →
Fam71d →
Vti1b ←
78.09
79.00
79.91
Chromosome 12 Location (Mbp)
Variant Type
indel
snp
SV

Supplemental Figure 5. Variant LOD scores across significant bQTL on chromosome 3 for week four mean 30% ethanol choice (W4_30C). (A) SNP associations were estimated across the 95% Bayesian confidence interval identified for the significant bQTL, identified by dashed vertical lines. Top variants (purple) were identified as those with a LOD score within 1.5 of the highest score. Statistical thresholds identified by permutation analysis are indicate as horizontal dotted lines. (B) Known gene transcript annotations within this C.I. are identified. Prioritized interval genes from Table 2 are highlighted in purple. These include *Zfyve26* with a missense mutation; top candidate genes from Table 2 are highlighted in purple.

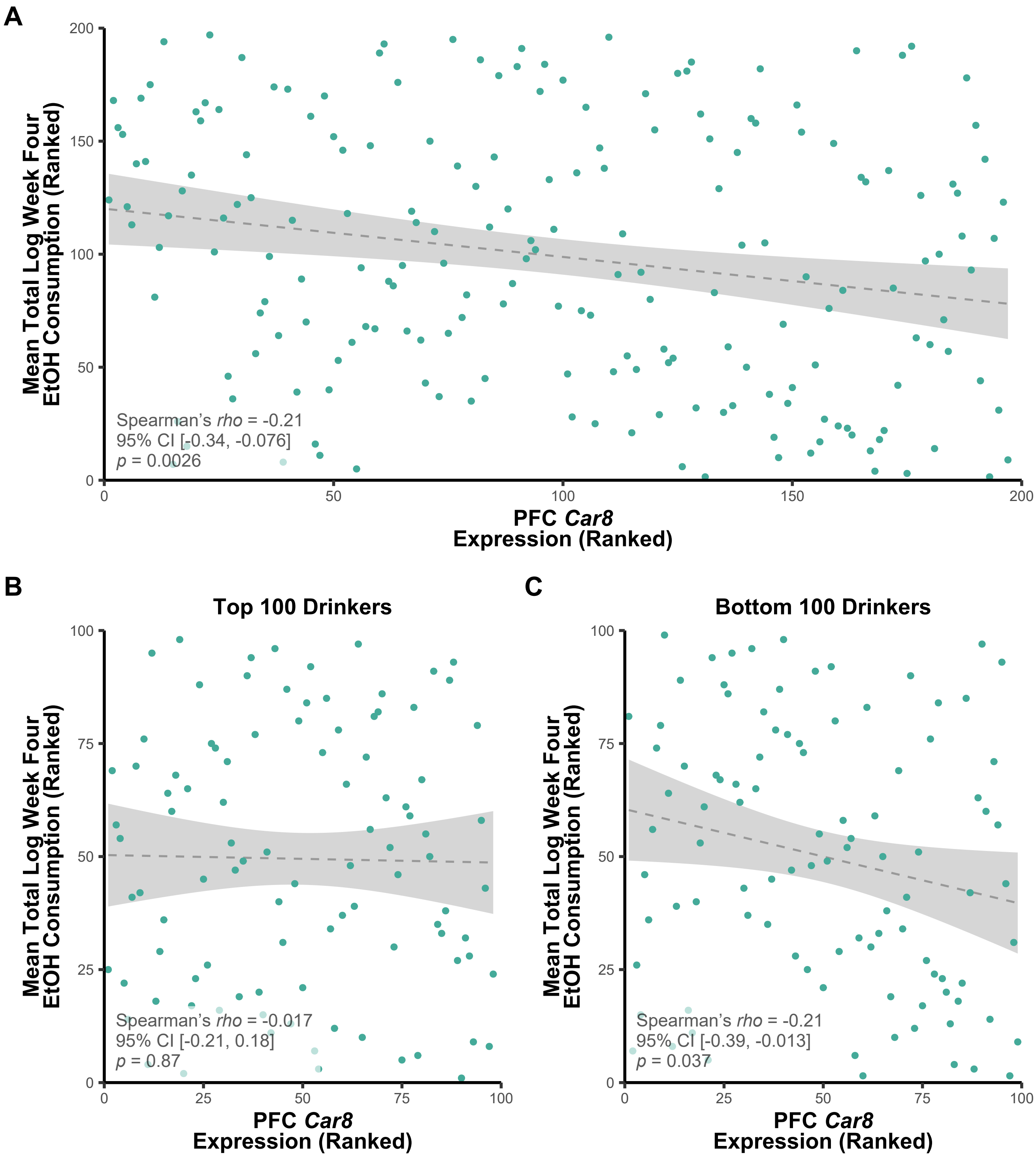

Supplemental Figure 6. PFC Car8 expression negatively correlates with voluntary ethanol consumption in male DO mice and in male and female BXD mice. Overall PFC Car8 expression data (A) as well as expression data from only the top 100 (B) and bottom 100 (C) drinkers were correlated with individual ethanol consumption in week four of the study. For the lowest 100 drinkers and the 200 total drinkers, this correlation was significant and negative; for the top 100 drinkers, the correlation was nonsignificant.

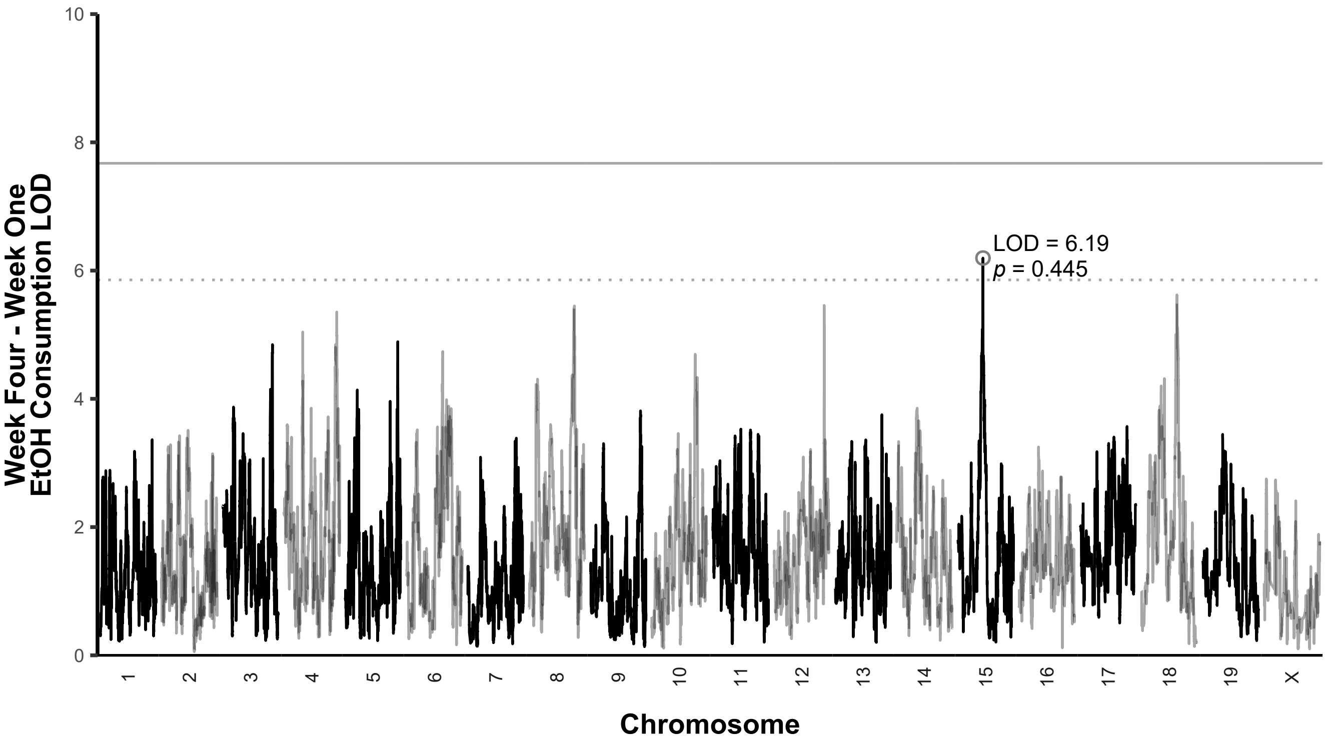

Supplemental Figure 7. Difference in week four versus week one ethanol consumption as a phenotype for behavioral QTL mapping. Change in ethanol consumption over time for each mouse was estimated by subtracting week one from week four mean ethanol consumption. A suggestive QTL was identified on Chromosome 15.
